# Prospective Evaluation of Side-Effects Following the First Dose of Oxford/AstraZeneca COVID-19 Vaccine among Healthcare Workers in Saudi Arabia

**DOI:** 10.3390/vaccines10020223

**Published:** 2022-01-30

**Authors:** Majid A. Darraj, Hesham M. Al-Mekhlafi

**Affiliations:** 1 Department of Internal Medicine, Faculty of Medicine, Jazan University, Jazan 45142, Saudi Arabia; 2 Medical Research Centre, Jazan University, Jazan 45142, Saudi Arabia; 3 Department of Parasitology, Faculty of Medicine and Health Sciences, Sana’a University, Sana’a 1247, Yemen

**Keywords:** COVID-19, vaccine, side-effects, healthcare workers, Saudi Arabia

## Abstract

Background: Several different types of vaccines have been developed for the prevention of coronavirus disease (COVID-19). Despite several local and systemic side-effects to COVID-19 vaccination reported, the vaccines are still considered the best intervention to tackle the spread of the virus and reduce the severity of COVID-19 infection. However, the reported side-effects continue to have a crucial role in public confidence in the vaccine and its acceptance. This study aimed to investigate the short-term side-effects reported by the healthcare workers (HCWs) in Saudi Arabia after receiving the first dose of the Oxford/AstraZeneca (ChAdOx1 nCoV-19) COVID-19 vaccine. Methods: A prospective cohort study was conducted among HCWs in the Jazan region of southwestern Saudi Arabia. Healthcare workers who had received the first dose of the COVID-19 vaccine and agreed to participate in the study were followed up for 3 weeks post vaccination through a weekly online survey. Information was collected on local and/or systemic side-effects reported following vaccine administration. Participants’ general and demographic information was also collected. Results: A total of 57.2% (250/437) of the HCWs who participated in this study reported at least one side-effect. Injection site pain and redness (80.0%), fever (73.2%), whole-body pain/fatigue (56.4%), and headache (48.8%) were the most commonly reported side-effects. Moreover, 12.4% of the participants who reported side-effects needed to see a physician, and only one female participant was admitted to the hospital. Multivariate regression analyses revealed that nationality (Saudi, adjusted odds ratio (aOR) = 3.65; 95% CI = 2.40, 5.55) and residency (Jazan governorate, aOR = 0.38; 95% CI = 0.15, 0.95) were the significant factors associated with reporting COVID-19 post-vaccination side-effects, while the number of reported side-effects was found to be significantly influenced by occupation (medical, aOR = 0.42; 95% CI = 0.26, 0.66; *p* < 0.001) and gender (female, aOR = 0.61; 95% CI = 0.38, 0.97; *p* = 0.038). Conclusions: Findings of the present study support the safety of the Oxford/AstraZeneca COVID-19 vaccine among HCWs in Saudi Arabia. All the reported side-effects were mild-to-moderate side-effects. The findings may help convince vaccine-hesitant individuals and skeptics to accept the COVID-19 vaccine.

## 1. Introduction

The novel coronavirus (COVID-19), caused by severe acute respiratory syndrome coronavirus 2 (SARS-CoV-2), was declared a global pandemic on 11 March 2020 [1]. Since then, most countries have taken precautions to stop COVID-19 from spreading in the hope of quickly developing safe and effective vaccines [2]. Extensive research has been condensed to bring a COVID-19 vaccine to market by late 2020 or early 2021, along with safety testing to prevent any serious side-effects [3]. Globally, Pfizer/BioNTech (BNT162b2) was the first vaccine authorized by the United States (US) Food and Drug Administration (FDA) in December 2020 [4], followed by Oxford/AstraZeneca being approved in January 2021 by the European Commission.

Many COVID-19 vaccine candidates have been developed by major global biopharmaceutical companies. The Pfizer/BioNTech Comirnaty vaccine was the first vaccine for COVID-19 listed on WHO’s Emergency Use Listing (EUL) on 31 December 2020. The AstraZeneca AZD1222 and SII Covishield vaccines developed by AstraZeneca/Oxford were given EUL on 16 February 2021. Moreover, the Johnson and Johnson’s Janssen Ad26.COV 2.S vaccine was listed for EUL on 12 March 2021, and the Moderna COVID-19 vaccine (mRNA 1273) was listed on 30 April 2021. The Sinopharm COVID-19 vaccine produced by Beijing Bio-Institute of Biological Products Co Ltd., a China National Biotec Group (CNBG) subsidiary, was listed for EUL on 7 May 2021. The Sinovac/CoronaVac was listed for EUL on 1 June 2021 [5].

These vaccines were developed using different technologies. The Pfizer/BioNTech BNT162b2 and Moderna mRNA 1273 COVID-19 vaccines are messenger RNA (mRNA)-based vaccines, which encode SARS-CoV-2 prefusion-stabilized full-length spike protein [6], with efficacy rates of 95% and 94.1%, respectively [7]. Likewise, the vaccines developed by Oxford/AstraZeneca and Johnson and Johnson are considered viral vector-based vaccines [8]. The Oxford/AstraZeneca vaccine consists of a replication-deficient chimpanzee adenoviral vector ChAdOx1 containing the SARS-CoV-2 structural surface glycoprotein antigen (spike protein; nCoV-19) gene, with an efficacy rate of 70% [9]. Both the Chinese vaccines (Sinopharm and Sinovac/CoronaVac) are inactivated vaccines, which use killed SARS-CoV-2 virus [8]. Overall, these vaccine types all met the necessary criteria for safety and efficacy as evaluated by the WHO [10].

However, these vaccines were linked to various adverse events, as concluded in clinical trials [11]. Moreover, several reports from different countries revealed that adverse events reported following the first and/or the second dose of COVID-19 vaccines included local side-effects, such as local pain at the site of injection, swelling, tenderness, redness, warmth, itch, and swollen armpit glands. They also included nonspecific systemic side-effects, such as headache, fever, fatigue, night sweats, chills and shivering, joint pain, muscle pain, nausea, vomiting, and decreased appetite [6,12,13,14,15,16,17]. Despite being very rare, a few clinical trials reported serious and life-threatening adverse events, such as vaccine-induced immune thrombocytopenia and thrombosis (VIIT), following the first dose of certain vaccines, mainly with viral vector vaccines [18,19]. Nonetheless, a recent interim analysis of surveillance data from over six million individuals found no association between VIIT and mRNA vaccines [20]. More information on adverse events is required at the global level to understand better the factors associated with different vaccine-specific adverse events, which could help guide further investigations and proper clinical management of the cases.

In Saudi Arabia, COVID-19 cases reached 547,845 confirmed cases by mid-October 2021 and 8758 total deaths [21]. The country started its COVID-19 vaccination program in December 2020 to stop the spread of COVID-19 [22]. Three vaccines are currently available in Saudi Arabia: Pfizer/BioNTech, Oxford/AstraZeneca, and Moderna COVID-19 vaccines [23]. About 45 million COVID-19 vaccine doses have been administered so far, with nearly 60% of the country’s total population receiving two doses of the vaccines [24]. While there are data on COVID-19 vaccine views expressed by the general population and their acceptability of vaccination [25], data on the attitude of healthcare workers (HCWs) toward the Oxford/AstraZeneca and Pfizer/BioNTech COVID-19 vaccines are limited [26]. However, little is known about the factors associated with COVID-19 vaccine refusal or hesitancy. Within this context, the present study aimed to identify short-term side-effects among HCWs in Saudi Arabia after receiving their first dose of the Oxford/AstraZeneca COVID-19 vaccine (ChAdOx1 nCoV-19).

## 2. Materials and Methods

### 2.1. Study Settings

A prospective cohort study was conducted among HCWs in the Jazan region of Saudi Arabia from May through June 2021. All vaccinated HCWs who had received their first dose of vaccination with the Oxford/AstraZeneca vaccine were followed up for 3 weeks post vaccination through a weekly online survey where the participants were asked to self-report any symptoms they developed. Those who had mild-to-moderate symptoms notified their clinic at the respective hospital via WhatsApp, SMS, or in person to set an appointment to be seen by a physician. A thorough assessment was performed and documented in their medical record for further follow-up by an infection control team for the specified 3 week period.

The target populations of the present study were all clinical (including physicians, nurses, medical technicians, and pharmacists) and nonclinical HCWs (including administrative and medical record professionals, workers, security, and nonmedical technicians) who were actively serving at participating private and public hospitals in Jazan region during the time of the study. The eligible participants were to have not received their first dose of COVID-19 vaccine before accepting the invitation to participate and filling in the questionnaire. Healthcare workers who were vaccinated before the study period were excluded. At the participating hospitals, an invitation message and information about the study objective and procedures were sent to all HCWs via their WhatsApp and email groups. Those who were eligible and willing to participate were listed, and their contact information was obtained. The participants were instructed to contact the survey team after they made appointments to get vaccinated.

Ethical approval was granted by the research ethics committee of Jazan University. A signed written informed consent was obtained from all participants prior to completing the survey. This procedure was approved by the research ethics committee. Participation in this survey was not compensated financially or by any other incentives.

### 2.2. Sample Size

The minimum sample size required for this survey was calculated according to Lwanga and Lemeshow [27]. As previous data on COVID-19 post-vaccination side-effects in the HCWs in Saudi Arabia were unavailable, a 0.50 assumed proportion was considered [28], with a 5% margin of error and 95% confidence level. Accordingly, 384 was yielded as the minimum number of participants required for this study. Considering a drop-out rate of 10%, the final sample size was determined as 422. During the study period, 485 eligible HCWs agreed to participate in the survey. 

### 2.3. Instrument and Data Collection

A structured self-administered questionnaire of this survey was created after extensive review of the literature on adverse events of various COVID-19 vaccines [11,12,29]. The questionnaire was designed using Google Forms and was written in English and Arabic. The final versions were reviewed by four different experts in the field to check for face and content validity. The questions addressed the demographic characteristics of respondents (age, sex, job category, marital status, residence, and nationality), medical anamnesis data (history of chronic diseases, and medical treatment taken regularly) and COVID-19-related anamnesis data (history of previous diagnosis with COVID-19, the type of vaccine they received, and vaccination date). The questionnaire also included questions for reporting short-term local and systemic side-effects associated with the COVID-19 vaccine, time of onset post vaccination, the duration of symptoms, any medication that were taken, doctor’s visits due to side-effects, and admission to the hospital. A thorough list of potential side-effects, covering the most common symptoms reported by previous studies, was provided in the questionnaire. Moreover, an open section was also provided for reporting other unlisted symptoms which might have been experienced by the respondents. 

Participants who made appointments to get vaccinated and notified the survey team were followed up. The survey’s link was sent to the participants through WhatsApp and/or e-mail. All participants were asked to respond to the survey; thus, the survey team members contacted those who did not respond weekly to identify and verify their responses. Only those who had received the first dose of Oxford/AstraZeneca vaccine and submitted the required questionnaires during the follow-up period were included in the final analysis. Out of 485 eligible participants, 41 (8.5%) individuals were lost to follow-up during the study period. Therefore, 437 HCWs were included in the final analysis.

### 2.4. Data Analysis 

The data were exported from Google Forms (Mountain View, CA, USA) into Microsoft Excel (Redmond, WA, USA) and then exported into the Statistical Package for Social Sciences (SPSS) software version 21.0 (IBM, Inc., Armonk, NY, USA) for statistical analysis. For descriptive analysis, frequency and proportion were used to express the distribution of categorical variables while mean ± standard deviation (SD) or median (interquartile range, IQR) were used to present quantitative variables. Before analysis, all quantitative variables were assessed for normality by Shapiro–Wilk test. Pearson’s chi-square (χ^2^) test or Fisher’s exact test was used where appropriate to examine the association between reporting COVID-19 post-vaccination side-effects as the dependent variable and the independent variables (demographic and health background characteristics). Likewise, Mann–Whitney U and Kruskal–Wallis tests were used to assess the number of COVID-19 post-vaccination side-effects reported by the studied HCWs as an ordinal dependent variable according to the independent variables.

Moreover, multivariate logistic regression analysis was also used to investigate the factors associated with reporting of side-effects following the Oxford/AstraZeneca COVID-19 vaccine, which was coded as a dummy dependent variable (yes = 1 and no = 0). In addition, multivariate ordinal logistic regression analysis was performed to assess the factors associated with the number of side-effects following the vaccine. All variables that showed associations with *p* ≤ 0.25 in the univariate analyses were included in the multivariate logistic regression models [30]. Odds ratios (ORs) with a 95% confidence interval (CI) were computed for different analyses. Statistical significance was defined as a *p*-value of less than 0.05. Where applicable, *p*-values were adjusted for multiple comparisons using the sequential Bonferroni correction (Bonferroni–Holm) method [31].

## 3. Results

### 3.1. General Characteristics of Participants

A total of 437 HCWs who were vaccinated with the first dose of the Oxford/AstraZeneca COVID-19 vaccine participated in the study. The mean ± SD age of the participants was 33.6 ± 7.3 years; around two-thirds (64.8%, *n* = 283) of them were females, and 35.2% were males. Nurses were the dominant HCW group at 38.2% (*n* = 167), followed by medical record professionals (14.0%, *n* = 61). The participants were from three governorates, namely, Jazan, Sabia, and Abu Arish, with the majority (86.5%,  378/437) living in Jazan city. Participants who had been previously diagnosed with COVID-19 made up 12.6% (*n* = 55), most of whom were females (80.0%, 44/55). Only three participants (0.7) had a history of chronic disease, both diabetes mellitus (DM) and hypertension. Table 1 shows the general demographic and health characteristics of the participants.

### 3.2. Reported COVID-19 Vaccine Side-Effects

**Figure 1:**
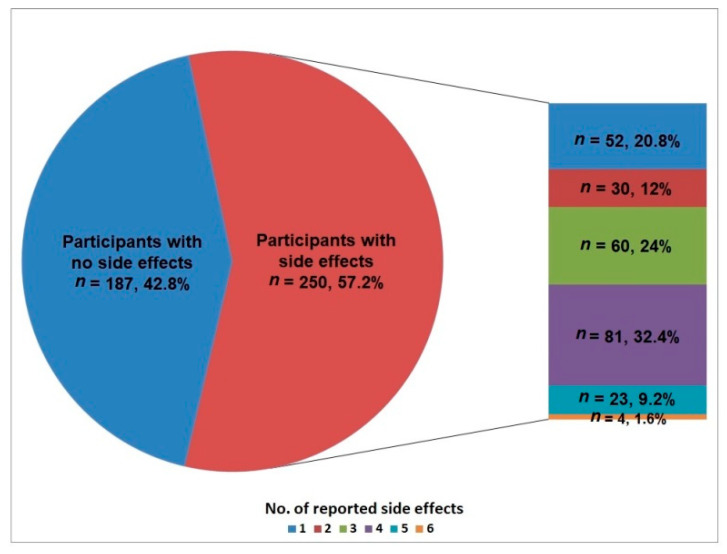
Total number of COVID-19 vaccine side-effects reported among the participants.

The results of this study showed that 57.2% (250/437) of the HCWs who participated in this study reported at least one COVID-19 post-vaccination side-effect. The median (IQR) number of the reported side-effects was 3.0 (2, 4). Figure 1 shows that most HCWs reported four side-effects (32.4%, 81/250), and 24% reported three side-effects, while six side-effects were reported by only 1.6%.

**Figure 2:**
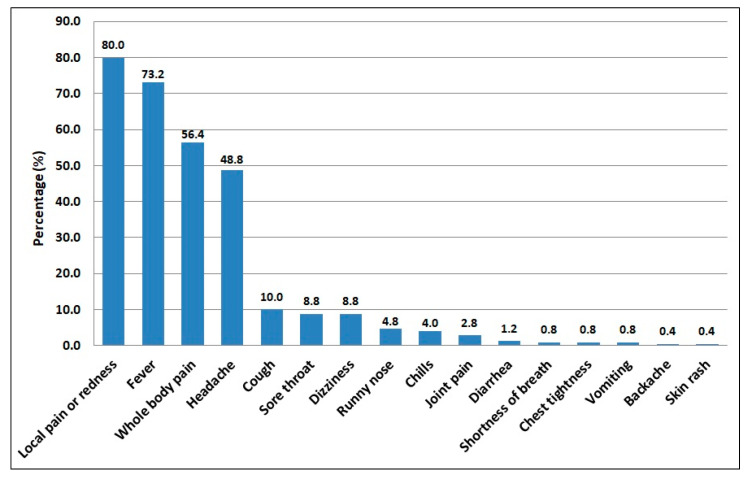
The frequency of COVID-19 vaccine side-effects reported among the participants (*n* = 250).

Among the 250 HCWs who developed COVID-19 post-vaccination side-effects, Figure 2 shows that pain and redness at the site of injection (80.0%) and fever (73.2%) were the most frequently reported side-effects, followed by whole-body pain/fatigue (56.4%) and headache (48.8%). On the other hand, the least frequently reported side-effects were diarrhea at 1.2%, shortness of breath, chest tightness, and vomiting at 0.8% each, and backache and skin rash at 0.4% each.

The distribution of different side-effects according to participants’ gender was investigated among the HCWs who developed COVID-19 post-vaccination side-effects (Table 2). The results showed that female HCWs had a significantly higher frequency of side-effects of dizziness (11.9% vs. 3.3%; χ^2^ = 5.236; *p* = 0.022) and chills (6.2% vs. 0.0%; χ^2^ = 5.859; *p* = 0.015) compared to male HCWs. On the other hand, the male HCWs had a significantly higher frequency of whole-body pain/fatigue (67.8%, 61/90) compared to their female peers (50.0%, 80/160) (χ^2^ = 7.403; *p* = 0.007). Nonetheless, these significant differences were not retained when the Bonferroni–Holm adjustment was applied.

### 3.3. Associations of the Reported COVID-19 Vaccine Side-Effects 

Table 3 shows the associations of COVID-19 vaccine side-effects with participants’ demographic and health background characteristics. The results showed comparable frequencies (*p* = 0.758) of HCWs who developed side-effects between female (56.5%, 160/283) and male (58.4%, 90/154) participants. Similarly, there was no significant difference in the frequency of side-effects according to participants’ age (*p* > 0.05). The side-effects were significantly more frequent among Saudi HCWs (73.8%, 138/187) compared to non-Saudis (44.8%, 112/250) (*p* < 0.001). Similarly, participants from the Jazan governorate had a significantly lower frequency of side-effects (54.2%) than that reported for participants from the Abu Arish (76.7%) and Sabia (75.9%) governorates (*p* < 0.05). Interestingly, medical HCWs had a significantly lower frequency of side-effects than nonmedical HCWs (51.0% vs. 65.1%; *p* = 0.003). After Bonferroni–Holm correction, only two variables, nationality (Saudi) and occupation (medical), showed a significant association with the reporting of side-effects, while the significance of residence (Jazan) was lost (Table 2).

Differences in the number of COVID-19 post-vaccination side-effects reported by the studied HCWs according to independent variables were examined, and the results are shown in Table 4. A Mann–Whitney U test showed that the number of side-effects was found to be significantly lower among the medical HCWs (median = 3; IQR = 1, 4) compared to the nonmedical HCWs (median = 4; IQR = 3, 4) (U = 5874; *p* < 0.001). On the other hand, the Kruskal–Wallis test results showed no significant difference in the number of side-effects according to age groups (H = 4.079; *p* = 0.245). Similarly, differences in the number of side-effects according to other variables were not significant (*p* > 0.05).

### 3.4. Multivariate Analyses of Factors Associated with the Reported COVID-19 Vaccine Side-Effects 

Table 5 presents the multivariate logistic regression analysis results for the factors associated with reporting side-effects following the first dose of the COVID-19 vaccine among the HCWs studied. All variables that showed associations with *p* ≤ 0.25 in the univariate analysis presented in Table 3 were included. The Hosmer–Lemeshow test, used for the inferential goodness-of-fit test, showed that the model fit the data well (χ^2^ = 4.909; *p* = 0.427). The results revealed that Saudi HCWs had a 3.65-fold increased risk of side-effects compared to non-Saudi HCWs (adjusted odds ratio (aOR) = 3.65; 95% CI = 2.40, 5.55). Moreover, HCWs who lived in the Jazan governorate had lower odds of reporting COVID-19 post-vaccination side-effects than those who lived in Abu Arish or Sabia (aOR = 0.38; 95% CI = 0.15, 0.95). However, the significant association of occupation with side-effects was not retained in the multivariate analysis (*p* = 0.067).

Furthermore, Table 6 shows the multivariate ordinal logistic regression analysis results for the factors influencing the number of COVID-19 vaccine side-effects reported by the studied HCWs. All variables that showed differences with *p* ≤ 0.25 in the univariate analysis presented in Table 4 were included in the model. The model was of good fit and statistically significant (χ^2^ = 19.672, *p* < 0.001). The number of reported side-effects was found to be significantly influenced by the occupation and gender of HCWs. The results showed that, holding other variables unchanged, medical HCWs were less likely to report a higher number of side=effects than their nonmedical peers (aOR = 0.42; 95% CI = 0.26, 0.66; *p* < 0.001). Similarly, female participants were less likely to report a higher number of side=effects than male participants (aOR = 0.61; 95% CI = 0.38, 0.97; *p* = 0.038).

### 3.5. Duration and Management of the Reported COVID-19 Vaccine Side-Effects

Among the HCWs who developed COVID-19 post-vaccination side-effects, 93.6% (234/250) of the reported side-effects occurred on the day of receiving the vaccine (Day 0). In comparison, 5.6% and 0.8% of the reported side-effects occurred Day 1 and Day 2 post vaccination (the second and third post-vaccination days), respectively. The duration of reported side-effects was 2–3 days for 53.2% (*n* = 133) and 4–5 days for 26.0% (*n* = 65), while the side-effects lasted for more than 7 days for only 2.4% (*n* = 6) of the subset of participants who reported side-effects. Moreover, 12.4% (*n* = 31) of those who developed side-effects visited a doctor, and medication for post-vaccination side-effects was taken by 66.4% (*n* = 166). Only one female participant (0.4%) was admitted to the hospital (Table 7).

## 4. Discussion

This study aimed to identify short-term side-effects following the Oxford/AstraZeneca COVID-19 vaccine among Saudi Arabian HCWs in Jazan region. It is essential to assure COVID-19 vaccine recipients at this critical stage of the vaccination campaigns by collecting evidence-based data concerning the vaccines’ side-effects, particularly if they are transient or temporary. Such evidence can alleviate fears and encourage the completion of the two-dose vaccination series [32].

The present study showed that 57.2% (250/437) of the studied HCWs reported at least one side-effect following the administration of the first dose of the Oxford/AstraZeneca COVID-19 vaccine. This finding is lower than recent findings reported in the Jazan region among the general population aged between 18 and 70 years who received the same type of COVID-19 vaccine (66.2%; 255/385) [33]. In a study conducted in Abha, Aseer Region, southwestern Saudi Arabia among 167 individuals aged 18 years and older who received the Oxford/AstraZeneca vaccine, 74.3% reported side-effects [29]. In contrast, a study among 1592 individuals in Dhahran City, northeastern Saudi Arabia, reported a lower incidence (34.7%) of side-effects following the Oxford/AstraZeneca vaccine [34]. Interestingly, Alghamdi et al. [35] found that adverse events and severity following the first dose of Oxford/AstraZeneca vaccine were more common in HCWs than non-HCW participants. Indeed, this difference could be attributed to psychosocial factors, as HCWs may be more sensitive to side-effects due to their health education than non-HCWs [36]. Moreover, due to their high occupational exposure to COVID-19, the likelihood of a previous asymptomatic COVID-19 infection among HCWs may lead to a stronger immune response compared to the general population [35]. Individuals who had been previously diagnosed with COVID-19 were more likely to report more side-effects after the first dose of the COVID-19 vaccine [15,37]. However, the present study found no considerable association between the development of side-effects and previous COVID-19 infection among the studied HCWs, and this agrees with previous studies in Saudi Arabia [33,38].

On the other hand, recent studies conducted in other countries reported higher frequencies of side-effects following the Oxford/AstraZeneca COVID-19 vaccine among different population groups. For instance, 97.8% of 197 Jordanian HCWs who received the first or the second dose of the Oxford/AstraZeneca COVID-19 vaccine reported some side-effects [39]. Likewise, another study from Poland showed that 96.5% of 705 participants reported at least one post-vaccination side-effect [40]. A slightly higher frequency of COVID-19 post-vaccination side-effects among HCWs was also reported in India (65%, 3556/5396) [41]. These differences could be attributed to different factors, including the incidence of COVID-19, the time these studies were conducted in relation to the pandemic waves and stages, and other demographic and health characteristics of the studied populations.

The side-effects can be local, such as injection site pain or redness, which was the most frequent side-effect reported by the studied participants, or systemic, including all remaining side-effects. Of these, fever was the second most frequently reported side-effect. The most common side-effects in the present study were injection site pain and redness, fever, fatigue, headaches, cough, sore throat, and dizziness. Less common side-effects were diarrhea, shortness of breath, chest tightness, vomiting, backache, and skin rash. These findings are similar to those reported among the general population in Saudi Arabia [33,38]. In comparison with studies conducted elsewhere, the reported frequencies of these side-effects were slightly higher than those reported by similar studies among HCWs in Jordan [39] and India [41]. On the other hand, higher frequencies of these side-effects were reported among the general population in Poland [40]. The present study found no severe complications linked to the first dose of the Oxford/AstraZeneca COVID-19 vaccine, and this agrees with previous studies in Saudi Arabia [29,33]. Nonetheless, serious adverse events such as severe allergic reaction, cardiac arrest, cerebral venous sinus thrombosis, and pulmonary embolism associated with the COVID-19 vaccine have been reported worldwide, including in Saudi Arabia [42,43].

In the current study, 93.3% of the side-effects occurred on the day of vaccination, and 35.6% (89/250) of the symptoms lasted for more than 3 days, which is consistent with the findings reported in other similar studies [33,39]. In the current survey, about two-thirds (66.4%, 166/250) of participants took medication (mainly analgesics) to relieve the side-effects. Taking analgesics to alleviate the side-effects associated with the COVID-19 vaccine is common among both HCWs and non-HCW populations in Saudi Arabia [35]. Moreover, only 12.4% of the participants needed to see a physician due to side-effects from the vaccines, and only one female participant was admitted to the hospital. This strongly supports the safety of these vaccines.

Regarding associations of post-vaccination side-effects with some demographic and health variables, the present study found that nationality and residence of the participants were the significant factors associated with the reporting of side-effects. Saudi HCWs had 3.65 times the odds of reporting side-effects compared to non-Saudi HCWs. This might be attributed to the perception of vaccine safety or the psychological and immunological status of the participants. Previous studies found that non-Saudi participants were more inclined to accept the COVID-19 vaccine [22]. They were also more likely to increase their physical activity during COVID-19 quarantine and mobility restrictions than their Saudi counterparts [44]. Moreover, the present study found that HCWs who lived in the Jazan governorate had lower odds of reporting COVID-19 post-vaccination side-effects than those who lived in Abu Arish or Sabia. While the explanation for this association is unknown, it might be attributed to the fact that the majority (86.5%) of the participants involved in this study were from the Jazan governorate.

Furthermore, the present study showed that medical HCWs (including nurses, physicians, medical technicians, and pharmacists) had lower odds of reporting post-vaccination side-effects than nonmedical HCWs (including administrative and medical record professionals, workers, security, and nonmedical technicians). However, this association was not retained when logistic regression analysis was applied. Interestingly, the present study demonstrated that medical HCWs were likely to report a higher number of side-effects than the nonmedical HCWs. These findings could be attributed to the higher level of medical knowledge about vaccine safety among the medical HCWs that would enhance their ability to identify and differentiate the symptoms.

The present study found comparable frequencies of reporting side-effects for male and female participants, in agreement with previous studies [39]. However, this finding contradicts that reported by Alhazmi et al. [33], who found a higher incidence of side-effects among female participants than male participants. In contrast, a study among 330 individuals in southwestern Saudi Arabia reported a higher incidence of side-effects in males than females following Pfizer/BioNTech and Oxford/AstraZeneca vaccination [29]. Interestingly, when ordinal logistic regression analysis was applied in the present study, the female gender was identified as a significant factor associated with reporting a lower number of side-effects compared to the male gender. This finding is consistent with recent studies conducted among 1592 individuals in Dhahran City, northeastern Saudi Arabia [34]. However, the finding is inconsistent with that of other reports from Saudi Arabia [38,45] and elsewhere [14,15,46], which suggested that female gender was a significant factor for the development of more severe and a higher number of COVID-19 post-vaccination adverse events. However, findings on the gender-related differences of COVID-19 vaccine side-effects are still inconclusive.

Indeed, previous studies have suggested a gender-based difference in reporting adverse events following various viral and bacterial vaccines [47,48]. It is found that females typically mount stronger inflammatory, antibody, and cell-mediated immune responses to vaccines when compared with males, and this might explain the sex-based differences in reactions and immunogenicity toward vaccinations [46]. Furthermore, behavioral, genetic, and hormonal factors might also underlie the gender-based variation in adverse events following vaccination [46,48,49]. In addition, a few studies have reported significant associations between the development of post-vaccination side-effects and age. Studies among HCWs in Jordan [39], Germany [14], and Slovakia [50] found that young adult participants had a significantly higher incidence of COVID-19 vaccine side-effects compared to their older age counterparts, and this has been attributed to the fact that immune responses gradually weaken with age. Nevertheless, in agreement with some studies conducted in Saudi Arabia [33] and elsewhere [51], the present study did not find such an association.

Some limitations of the current study should be acknowledged to be considered when interpreting the findings. First, this study used an online self-administered questionnaire instead of face-to-face interviews, which may result in reporting bias. However, the HCWs participants are expected to have adequate knowledge about the vital importance of the COVID-19 vaccine and its associated adverse events. Second, this study reported only short-term side-effects, while the vaccines’ intermediate and long-term side-effects were not studied. Similarly, only side-effects following the first dose of the vaccine were studied, while the side-effects following the second dose of the vaccine were not evaluated. Furthermore, the study was also predominated by Oxford/AstraZeneca recipients with only a few Pfizer/BioNTech recipients. This could have been due to the shortage of the BioNTech vaccine during the study duration and the relatively small sample size; thus, further multicenter studies with larger sample sizes are warranted to ascertain the safety profile of different COVID-19 vaccines approved for use in the country.

## 5. Conclusions

The present study provides important information on the side-effects following COVID-19 vaccination among healthcare workers in Saudi Arabia. The findings showed that most participants reported pain at the injection site, fever, fatigue, and headache. Moreover, nationality (Saudi) and residence (Jazan) were identified as the significant factors associated with the reporting of side-effects following the first dose of the Oxford/AstraZeneca COVID-19 vaccine, while gender (female) and occupation (medical) were significantly associated with reporting a lower number of side-effects. The findings revealed that all the COVID-19 post-vaccination side-effects were mild to moderate, and only a small portion of vaccine recipients (12.4%) needed to see a physician, while only one participant (0.4%) admitted to the hospital due to those side-effects. Therefore, the current study’s findings support the vaccine’s safety and provide important baseline data to increase healthcare workers’ and the general community’s awareness of the expected side-effects following COVID-19 vaccines. This might help convince the vaccine-hesitant individuals and skeptics to accept the COVID-19 vaccine.

## Figures and Tables

**Table 1:** General demographic and health characteristics of the participants (*n* = 437).

Characteristics		*n* (%)
Age group (years)	20–30	169 (38.7)
	31–40	202 (46.2)
	41–50	50 (11.4)
	>50	16 (3.7)
Nationality	Saudi	187 (42.8)
	Non-Saudi	250 (57.2)
Gender	Females	283 (64.8)
	Males	154 (35.2)
Marital Status	Married	250 (57.2)
	Single	187 (42.8)
Residence	Jazan	378 (86.5)
	Sabia	29 (6.6)
	Abu Arish	30 (6.9)
Occupation	Nurse	167 (38.2)
	Medical record professional	61 (14.0)
	Administrative professional	52 (11.9)
	Workers (such as cleaning staff, drivers, storekeepers)	45 (10.3)
	Physician	39 (8.9)
	Technician—medical	37 (8.5)
	Security	18 (4.1)
	Technician—nonmedical	16 (3.7)
	Pharmacist	2 (0.5)
Diagnosed previously with COVID-19	Yes	55 (12.6)
	No	382 (87.4)
History of chronic diseases	DM and hypertension	3 (0.7)
	No	434 (99.3)

**Table 2:** Distribution of COVID-19 vaccine side-effects reported among the healthcare worker participants according to gender (*n* = 250).

Symptoms	Male (*n* = 90)	Female (*n* = 160)	Total (*n* = 250)	χ^2^ (*p*)
Pain or redness at the site of injection	72 (80.0)	128 (80.0)	200 (80.0)	0.001 (0.999)
Fever	65 (72.2)	118 (73.8)	183 (73.2)	0.069 (0.793)
Whole body pain/fatigue	61 (67.8)	80 (50.0)	141 (56.4)	7.403 (0.007)
Headache	51 (56.7)	71 (44.4)	122 (48.8)	3.483 (0.062)
Cough	13 (14.4)	12 (7.5)	25 (10.0)	3.086 (0.079)
Sore throat	12 (13.3)	10 (6.2)	22 (8.8)	3.601 (0.058)
Dizziness	3 (3.3)	19 (11.9)	22 (8.8)	5.236 (0.022)
Runny nose	6 (6.7)	6 (3.8)	12 (4.8)	1.072 (0.301) ^†^
Chills	0 (0.0)	10 (6.2)	10 (4.0)	5.859 (0.015) ^†^
Joint pain	2 (2.2)	5 (3.1)	7 (2.8)	0.172 (0.587) ^†^
Diarrhea	1 (1.1)	2 (1.2)	3 (1.2)	0.009 (0.735) ^†^
Shortness of breath	0 (0.0)	2 (1.2)	2 (0.8)	1.134 (0.549) ^†^
Chest tightness	0 (0.0)	2 (1.2)	2 (0.8)	1.134 (0.549) ^†^
Vomiting	0 (0.0)	2 (1.2)	2 (0.8)	1.134 (0.549) ^†^
Backache	1 (1.1)	0 (0.0)	1 (0.4)	1.785 (0.630) ^†^
Skin rash	0 (0.0)	1 (0.6)	1 (0.4)	0.565 (0.710) ^†^

χ^2^, chi-square test statistic. Significant difference between the two groups (unadjusted *p* < 0.05). ^†^ Fisher’s exact test statistic.

**Table 3:** Associations of the reported COVID-19 vaccine side-effects with the participants’ background characteristics (*n* = 437).

Characteristic	Reported Side-Effects		OR (95% CI)	*p*
	Yes (*n* = 250)	No (*n* = 187)		
Age group (years)				
20–30 (*n* = 169)	94 (55.6)	75 (44.4)	1	
31–40 (*n* = 202)	115 (56.9)	87 (43.1)	1.06 (0.70, 1.60)	0.801
41–50 (*n* = 50)	29 (58.0)	21 (42.0)	1.10 (0.58, 2.09)	0.766
>50 (*n* = 16)	12 (75.0)	4 (25.0)	2.39 (0.74, 7.72)	0.134
Gender				
Female (*n* = 283)	160 (56.5)	123 (43.5)	0.93 (0.62, 1.38)	0.701
Male (*n* = 154)	90 (58.4)	64 (41.6)	1	
Nationality				
Saudi (*n* = 190)	138 (73.8)	49 (26.2)	3.47 (2.30, 5.23)	<0.001 *
Non-Saudi (*n* = 254)	112 (44.8)	138 (55.2)	1	
Marital status				
Married (*n* = 255)	145 (57.2)	105 (42.0)	1.08 (0.74, 1.58)	0.699
Single (*n* = 189)	105 (56.1)	82 (43.9)	1	
Residence				
Jazan (*n* = 378)	205 (54.2)	173 (45.8)	0.36 (0.15, 0.86)	0.017
Sabia (*n* = 29)	22 (75.9)	7 (24.1)	0.96 (0.29, 3.18)	0.924
Abu Arish (*n* = 30)	23 (76.7)	7 (23.3)	1	
Occupation				
Medical (*n* = 245)	125 (51.0)	120 (49.0)	0.56 (0.38, 0.82)	0.003 *
Nonmedical (*n* = 192)	125 (65.1)	67 (34.9)	1	
Diagnosed previously with COVID-19				
Yes (*n* = 55)	28 (50.9)	27 (49.1)	0.75 (0.43, 1.32)	0.313
No (*n* = 382)	222 (58.1)	160 (41.9)	1	

OR, odds ratio; CI, confidence interval. Significant association (unadjusted *p* < 0.05). * Significant association (using the Bonferroni–Holm correction for multiple comparisons).

**Table 4:** Number of side-effects reported by the participants following the first dose of COVID-19 vaccine according to the participants’ background characteristics (*n* = 250).

Characteristic	No. of Reported Side-Effects	Statistics	*p*
	Median (IQR)		
Age group (years)		H = 4.079	0.245
20–30 (*n* = 94)	3 (2, 4)		
31–40 (*n* = 115)	3 (2, 4)		
41–50 (*n* = 29)	3 (1, 4)		
>50 (*n* = 12)	2 (1, 3)		
Gender		U = 6366	0.117
Female (*n* = 90)	3 (2, 4)		
Male (*n* = 160)	3 (2, 4)		
Nationality		U = 7643	0.878
Saudi (*n* = 138)	2 (3, 4)		
Non-Saudi (*n* = 112)	2 (3, 4)		
Marital status		U = 7475	0.802
Married (*n* = 145)	3 (2, 4)		
Single (*n* = 105)	3 (2, 4)		
Residence		H = 2.125	0.346
Jazan (*n* = 205)	3 (2, 4)		
Sabia (*n* = 22)	3 (2, 4)		
Abu Arish (*n* = 23)	4 (3, 4)		
Occupation		U = 5874	<0.001 *
Medical (*n* = 125)	3 (1, 4)		
Nonmedical (*n* = 125)	4 (3, 4)		
Diagnosed previously with COVID-19		U = 3062	0.896
Yes (*n* = 28)	3 (3, 4)		
No (*n* = 222)	3 (2, 4)		

IQR, interquartile range; U, Mann–Whitney U statistic; H, Kruskal–Wallis test statistic. Significant difference (unadjusted *p* < 0.05). * Significant difference (using the Bonferroni–Holm correction for multiple comparisons).

**Table 5:** Multivariate analysis of factors associated with reporting of side-effects following COVID-19 vaccine among the participants (*n* = 437).

Variable	aOR	95% CI	*p*
Nationality (Saudi)	3.65	2.40, 5.55	<0.001 *
Residence (Jazan)	0.38	0.15, 0.95	0.038 *
Occupation (medical)	0.67	0.43, 1.02	0.062

aOR, adjusted odds ratio; CI, confidence interval. * Significant association of COVID-19 vaccine side-effects (*p* < 0.05).

**Table 6:** Results of ordinal logistic regression for the factors associated with the number of reported side-effects following COVID-19 vaccine (*n* = 250).

Variable	aOR	95% CI	*p*
Age (year)	0.97	0.94, 1.49	0.093
Gender (female)	0.61	0.38, 0.97	0.038 *
Occupation (medical)	0.42	0.26, 0.66	<0.001 *

aOR, adjusted odds ratio; CI, confidence interval. * Significant association (*p* < 0.05).

**Table 7:** The onset, duration, and management of COVID-19 vaccine side-effects reported among the healthcare workers participated in the study (*n* = 250).

Onset and Duration of Side-Effects	Male (*n* = 90)	Female (*n* = 160)	Total (*n* = 250)	χ^2^ (*p*)
Onset				0.172 (0.918)
Day 0	84 (93.3)	150 (93.8)	234 (93.6)	
Day 1	5 (5.6)	9 (5.6)	14 (5.6)	
Day 2	1 (1.1)	1 (0.6)	2 (0.8)	
Duration of symptoms				5.196 (0.268)
One day	8 (8.9)	20 (12.5)	28 (11.2)	
2–3 days	46 (51.1)	87 (54.4)	133 (53.2)	
4–5 days	30 (33.3)	35 (21.9)	65 (26.0)	
6–7 days	4 (4.4)	14 (8.8)	18 (7.2)	
More than 7 days	2 (2.2)	4 (2.5)	6 (2.4)	
Medication taken for side-effects	62 (68.9)	104 (65.0)	166 (66.4)	0.390 (0.532)
Doctor’s visit due to side-effects	10 (11.1)	21 (13.1)	31 (12.4)	0.215 (0.643)
Hospitalization due to side-effects	0 (0.0)	1 (0.6)	1 (0.4)	0.565 (0.641) ^†^

χ^2^, chi-square test statistic; ^†^ Fisher’s exact test statistic.

## Data Availability

The data that support the findings of this study are available from the authors upon reasonable request.
